# Factors Influencing Burden in Spouse-Caregivers of Patients with Chronic-Acquired Brain Injury

**DOI:** 10.1155/2020/6240298

**Published:** 2020-06-30

**Authors:** Stefania Laratta, Lucia Francesca Lucca, Paolo Tonin, Antonio Cerasa

**Affiliations:** ^1^S. Anna Institute and Research in Advanced Neurorehabilitation (RAN), Crotone, Italy; ^2^Institute for Biomedical Research and Innovation, National Research Council, 87050 Mangone, Italy

## Abstract

In this cross-sectional study, we assess associated factors of burden in spouse-caregivers of patients with acquired brain injury (ABI) in the chronic phase. 35 spouse-caregivers (71% female, mean age ± SD: 55.7 ± 11.1y) of patients with mild/moderate ABI (29% female, mean age ± SD: 57.5 ± 10.7y), admitted to the intensive rehabilitation unit of the Institute S. Anna (Crotone, Italy) between January 2013 and December 2017, were contacted 2 years postinjury and asked to complete a series of questionnaires. The outcome measure was the Caregiver Burden Inventory (CBI) test, while several demographical and clinical data were considered as predictive factors. Two years after injury, a high level of burden was reported in 34.2% of spouse-caregivers. Stepwise multiple linear regression analyses revealed that caring for a patient with more severe disability (as measured by the Barthel Index scale) and the family life cycle (from the initial phase of engagement to marriage with adult children) explain the vast majority of variance for higher caregiver burden. The functional clinical status and the stages through which a family may pass over time were identified as areas in which the spouse-caregiver of ABI patients experienced high levels of burden in the chronic phase.

## 1. Introduction

Acquired brain injury (ABI) is caused by head trauma or a cerebrovascular event, which can lead to cognitive, emotional, affective, and behavioral changes, thus invalidating patient's feelings and family relationships. The support of family caregivers given to their relatives with ABI has an important impact on clinical outcomes [1], and family-focused therapy has proven to be effective [2]. In addition, family care can reduce the requirements and obligations of professional caregivers and the formalized healthcare system [3].

Assisting a person with cognitive disabilities as a result of brain damage has a large impact on the emotional and physical health of the caregiver. This type of caregiving can drastically influence the balance in reciprocal relationships, thus increasing emotional distress and changes in the perception of recovery or rehabilitation outcomes [3–5]. Zarit and colleagues [6] defined burden as “the extent to which caregivers perceive the adverse effect that caregiving has on their emotional, social, financial, and physical functioning.”

Moderate levels of burden have been reported in caregivers of ABI patients ranging from 33% to 56%, with more severe levels reported in 10%-20% of cases [3, 7, 8]. Three categories of problems have been proposed as being more stressful for caregivers: (1) the disruption in interpersonal relationships (i.e., poor social network and social isolation) [9, 10]; (2) the patient's overall disability [3]; and (3) patient's overall functioning (i.e., mood disorders, cognitive deficits, and noncompliance with therapy) [11, 12]. In addition, being the main caregiver may have a more negative impact on spouses than parents [13], and time since injury might influence the level of burden and the predictors of burden [14].

However, poor family functioning has been reported as one of the factors relating to negative caregiving outcomes for brain injury patients [15]. Generally, every family undergoes significant changes over time in terms of roles, and expected events trigger the transition from one phase of the life cycle to another. In contrast, unexpected severe events, such as brain injuries, can interfere with normal transitions, interfering with the life cycle and negatively impacting on family life [15].

The life cycle of the family is a theoretical reference model, which originated from the field of systemic-relational psychotherapy [16]. According to this model, there are specific phases or evolutionary stages of family development: (1) creation of the conjugal couple; (2) family with a young child; (3) family with an adolescent child; (4) family with an adult child; and (5) family in old age. Family life is characterized by a rhythmic path, marked by the passage from one stage to another (family transitions). In each stage, individual family members are involved in different relationships, on different levels and with different developmental tasks. The transition from one stage to another in the life cycle of the family is favoured, but not necessarily determined, by expected (birth, marriage) or unexpected (i.e., divorce, illness, and death) critical events. Generally, in the first phases of the family life cycle, parents are totally focused on the children and are gratified by their growth, whereas when they become adults, parents can experience loneliness [17]. A study on cardiological diseases suggested that the presence of children in the family could be a supportive factor, with the patient showing a more moderate or “discreet” experience of symptoms [18]. Otherwise, the absence of children at home may be one of the causes of the experience of more “ostentatious” symptoms [18]. Instead, the evaluation of the impact of the family life cycle in ABI patients is still unclear.

Most studies have investigated the caregiver burden in the acute phase, whereas there is a paucity of data on the chronic phase. It is, thus, essential to determine the factors influencing the psychological health of carers over the course of the caregiving trajectory. Here, the aim was to determine whether there are specific demographical or clinical variables influencing the burden experienced by spouses of patients with mild/moderate ABI.

## 2. Materials and Methods

### 2.1. Participants

**Figure 1:**
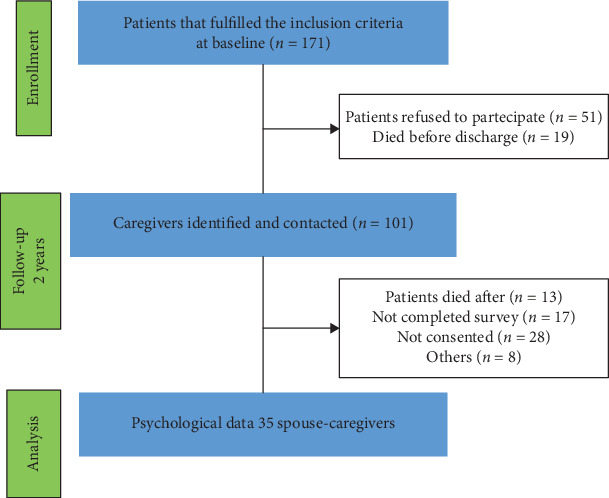
Flow diagram of participant recruitment and participation in the study.

The study was realized on spouses of patients with acute traumatic or vascular brain injury who had consecutively been admitted to the intensive rehabilitation unit (IRU) of the Institute S. Anna (Crotone, Italy) between January 2013 and December 2017. We only included patients without severe cognitive deficits as measured by MMSE (<23), recorded at the admission in IRU. At the time of inclusion, patients were asked to name their spouse-caregivers who could be contacted. Patients who agreed to involve the caregivers gave written informed consent. From an initial cohort of 171 ABI patients, 51 refused to participate and 18 died before discharge. Of the sample of 101 former pairs of patients and spouses contacted at 2 years follow-up, we included adults (age > 18 years) without a premorbid history of psychiatric diseases. Finally, thirty-five spouse-caregivers fulfilled the surveys and were finally included in the study (see Figure 1).

Patients who agreed to involve the caregivers gave written informed consent, approved by the Ethical Committee of the Central Area Regione Calabria of Catanzaro, according to the Helsinki Declaration.

### 2.2. Design and Procedure

This was a cross-sectional study. After two years from the event, if the caregivers gave oral agreement to participate, they were contacted by telephone, referring to the period 2013-2017. The surveys were sent by post or email. All participating completed a series of data collection referred either to the patients (at the time of IRU admission) or caregivers. The included variables were as follows:

- Demographical data including characteristics of family: age; sex; educational qualification (1=primary/2=secondary/3=high school/4=university); job activity (0=No/1=Yes) before traumatic event; duration of their relationship (years); number of children; family life cycle (1=initial engagement/2=spouse without child/3=spouse with a young child/4=spouse with adult child); spirituality (0=No/1=Yes); and frequency of participation in religious activities (1=no activity/2=sparse/3=often/4=very often)
- Clinical data: Barthel Index [19]; ABI phenotype (1=vascular, 2=traumatic); brain lesion localization (1=parietal/2=temporal/3=frontal/4=occipital); and side lesion (1=left/2=right).

All these variables have been included as predictor factors in the regression analysis. Moreover, in the assessment of the family life cycle, the adult child does not live with their families.

### 2.3. Outcome Measures

The main outcome was the Caregiver Burden Inventory (CBI) test. The CBI is a tool for assessing caregiver's care-load of patients with disabilities [20]. It is self-administered, divided into 5 sections, relating to their respective stressors: objective, psychological, physical, social, and emotional load. (1) The first factor dependent on the time required for care (items 1-5), which describes the load associated with the time restriction for the caregiver; (2) the evolutionary burden (items 6-10) refers to the perception of the caregiver to feel cut off from the expectations and opportunities of their peers; (3) the physical burden (items 11-14) describes feelings of chronic fatigue and somatic health problems; (4) the social burden (items 15-19) describes the perception of a role conflict; (5) and finally, the emotional burden (items 20-24) describes feelings towards the patient.

### 2.4. Statistical Analysis

Statistical analysis was performed using the Statistical Package for Social Science software (SPSS, v20.0, Chicago, IL, USA) for Macintosh. Assumptions for normality were tested for all continuous variables. Normality was tested using the Kolmogorov–Smirnov test. All variables were normally distributed. Simple regression (Spearman's *r*) was used to test the relationships among all variables, while stepwise multiple regression analysis was performed to evaluate the impact of demographic and clinical variables on CBI scores. For all tests, a *p* < 0.05 threshold was considered to be statistically significant.

## 3. Results

### 3.1. Clinical Data

The demographical characteristics of patients and their relative caregivers are reported in Tables 1 and 2. There are no differences in age and educational levels, whereas gender distribution is obviously different. Thirty-four percent of caregivers are married with a young child, whereas almost all are religious. As concerns burden levels, the majority of the caregivers experienced a high level (34.2%), whereas 20% reported a moderate level. The ABI patients were characterized by a mild clinical status strongly improved after the IRU period (*t* − value = −10.8; *p* level < 0.0001).

### 3.2. Regression Analysis

The simple regression analysis revealed a significant relationship among CBI scores and age of caregiver (*r* = 0.38; *p* level = 0.024), duration of relationship (*r* = 0.37; *p* level = 0.028), family life cycle (*r* = 0.43; *p* level = 0.01), Barthel Index (*r* = −0.36; *p* level = 0.03), and hemispheric lesion (*r* = −0.34; *p* level = 0.04). In other words, the increasing of age, the duration of relationship and severity status, as well as the presence of an adult child in the family, and lesions in the left side are variables associated with a higher level of caregiver burden.

**Figure 2:**
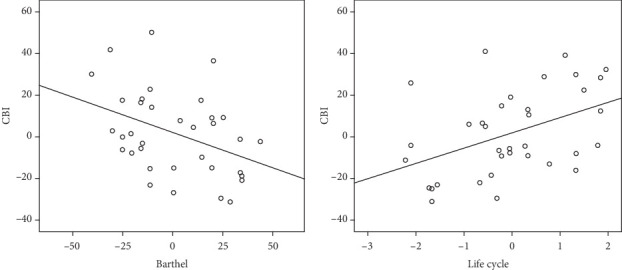
The functional status and the family life cycle factors explain the largest amount of variance in the caregiver burden of ABI patients.

The stepwise multiple regression analysis extracted two models that better predict the CBI scores. The first model only included the Barthel Index scores as the only factorable to explain alone 26% of the variability of the CBI scores (*r* = −0.51; *p* level = 0.005). Instead, the second predictive model includes the Barthel Index together with the family life cycle factor, that together explains 42% of the variability of the CBI (*r* = 0.64; *p* level = 0.01). Regression data also revealed a negative correlation between the Barthel Index and CBI scores and a positive correlation between the life cycle factor and CBI scores (Table 3; Figure 2). In synthesis, caring for a patient with more severe disability and living with the presence of an adult child in the family explain the vast majority of variance for higher caregiver burden.

## 4. Discussion

The occurrence of ABI is one of the main causes of permanent dependence. The family caregiver plays an important role in the recovery process, and, in some instances, they may even be the only source of support. It has been widely demonstrated that when the caregiver or close family member experiences a lower burden, less anxiety, and good health, this has a positive impact on the person with ABI [21, 22]. The burden in the acute phase is likely to be correlated with the patient's neurobehavioral problems [23] and level of functioning both in terms of stroke and head trauma [24, 25].

Our study emphasizes how brain injury continues to have an impact on daily life and negatively affects family carers. We showed that there is also a high level of burden in spouses of patients with mild/moderate ABI in the chronic phase. The highest burden was found to be related to patients with more severe injuries and as a function of the family life cycle. Indeed, families where the spouse of the patient with a brain injury had an adult child in the family were characterized by a higher level of burden.

### 4.1. The Impact of Disability on the Burden of Spouse-Caregivers

Because of the chronic nature of ABI, the care required is also permanent, and if the burden of this care is placed on the same person, this can impact negatively on their quality of life [26]. There are few studies investigating the caregiver burden in the chronic phase. Doser and Norub [14] demonstrated over a very long period (from 3-6 years) that the main factor influencing the burden of spouse-caregivers was the severity of the clinical status of the ABI patients. Bayen et al. [3] showed that one year after the injury, the caregiver burden was associated with dysexecutive functioning and the patients' overall disability. However, another study within a similar time frame demonstrated that the best predictor was the importance of the caregiver's needs and the percentage of needs met [24]. Our data agree with the vast majority of these studies, although the clinical assessment was performed using the Barthel Index, a scale developed to assess functional motor independence in patients with chronic disability.

### 4.2. The Impact of the Family Life Cycle on the Burden of Spouse-Caregivers

Brain injuries have long-term consequences on the functional status and psychosocial functioning [15, 27]. Although the data on caregivers' distress and burden are constantly increasing, less information is available on the role of family functioning. We found that the phases through which a family passes over time are related to the high burden experienced by caregivers. This is in agreement with the hypothesis that the family goes through phases that influence its functioning [28]. These dynamics may be particularly salient for families with disabilities because the caregiving demands may be out of synch with the family's life cycle phase. Indeed, Moore et al. [29] demonstrated that families with a father with traumatic brain injury were at substantial risk of being dysfunctional when they had young children.

The transition periods in the family life cycle of patients with ABI are when families reevaluate the appropriateness of their previous life structures in the face of new illness-related, developmental demands. Unfinished business from the previous phase can complicate or block movement through the transitions [30]. It is hard for families to find a compromise between members' individual developmental needs and the caregiving demands of a serious illness or disability. Some families then become frozen around the family organization at the time of the crisis, rather than being able to shift and reorganize to care for the chronic demands of an illness or disability [31]. During the chronic phase, the family needs to be able to care for the illness and still have energy, resources, and space for the patient and other family members. For example, the parents of an adolescent with cognitive disabilities may need to arrange social opportunities or help with grooming at a time when their other children could be leaving home. Similarly, disabled parents often need help from their children in ways that the life cycle model does not predict. Generally, when a disability is first revealed, there is a period of disequilibrium and a series of adjustments that need to be made by caregivers, and also by other family members [32].

In our study, we found that the final family stage (when the adult child had left home) is one of the main factors influencing the caregiver burden. This could be explained by the negative emotions experienced by taking care of a disabled spouse in presenile or senile age, hampered by a feeling of being alone with limited social support from family. Our findings suggest that special attention should be paid to how specific family functioning can influence diseases (and vice versa) according to their evolution and to the psychological demands they pose in specific phases of the life cycle [30]. This kind of evidence could help clinicians in early decision making such as treating patients with family counseling addressing intergenerational issues, also involving adult children.

### 4.3. Limitations

This work has some limitations. Firstly, a larger sample size would likely have been more representative of this heterogeneous neurological population. Secondly, we did not include other variables that could have better explained the caregiver burden. For instance, previous studies have highlighted the role of the caregivers' perception of patient depression or the evaluation of social support as the main factor significantly contributing to the caregiver burden [10, 12]. Finally, sexuality in the couple was only briefly referred to in this study; thus, it would be interesting to evaluate the impact of the changes in intimacy in future studies [33].

## 5. Conclusions

Two years after injury, we determined which factors affected the burden experienced by spouse-caregivers of patients with mild/moderate ABI. Our findings showed that taking care of ABI survivors is exacerbated by the worsening of disability and by the family life cycle [34]. Our findings may have several implications for the care management of ABI patients, also helping to identify partners who are at the greatest risk of pathological levels of burden.

## Figures and Tables

**Table 1:** Clinical and demographical characteristics of ABI patients.

	Variables	
Demographical	Age (years)	55.7 ± 11.1
	Sex (% f)	29% female
	Educational qualification	37% high school/17% university
	Job employment (Y/N)	71% No
Clinical	Barthel Index at admission	26.9 ± 21.4
	Barthel Index at discharge	61 ± 23.6
	ABI phenotype	(i) 57% vascular (ii) 43% traumatic
	Brain lesion localization	(i) 44% frontal lobe (ii) 34% temporal lobe (iii) 7% parietal lobe (iv) 15% occipital lobe
	Hemispheric lesion (% left)	46% left

**Table 2:** Demographical characteristics of spouse-caregivers.

Variables	
Age (years)	55.7 ± 11.1
Sex (% f)	71% female
Educational qualification	37% high school/23% university
CBI: high/moderate levels	34.2%/20%
Job employment (Y/N)	71% No
No. children	2 (0-4)
Life cycle	(i) 8% initial engagement (ii) 17% spouse without child (iii) 34% spouse with young child (iv) 14% spouse with adult child
Spiritual orientation (Y/N)	8% No
Religion commitment	(i) 28% often (ii) 34% very often

**Table 3:** Linear multiple regression analysis.

	No standardized coefficient	Standardized coefficient	*t*	Sig.
	*B*	Beta		
1. Model	55.584		5.569	0.000
Barthel	-0.473	-0.512	-3.043	0.005
2. Model	28.718		2.079	0.048
Barthel	-0.389	-0.421	-2.684	0.013
Family life cycle	0.696	0.404	2.575	0.016

## Data Availability

The data used to support the findings of this study are available from the corresponding author upon request.
